# Passively scanned, single‐fiber optical coherence tomography probes for gastrointestinal devices

**DOI:** 10.1002/lsm.23576

**Published:** 2022-06-16

**Authors:** David O. Otuya, Nicholas M. Dechene, Darina Poshtupaka, Seth Judson, Camella J. Carlson, Sarah K. Zemlok, Evan Sevieri, Peter Choy, Rachel E. Shore, Esmarline De León‐Peralta, Alissa A. Cirio, Tyler W. Rihm, Alexander A. Krall, Evangelia Gavgiotaki, Jing Dong, Sarah L. Silva, Aaron Baillargeon, Grace Baldwin, Anna H. Gao, Zachary Jansa, Amilcar Barrios, Emily Ryan, Nitasha G. M. Bhat, Indira Balmasheva, Anita Chung, Catriona N. Grant, Ara L. Bablouzian, Matthew Beatty, Osman O. Ahsen, Hui Zheng, Guillermo J. Tearney

**Affiliations:** ^1^ Wellman Center for Photomedicine Massachusetts General Hospital Boston Massachusetts USA; ^2^ Harvard Medical School Boston Massachusetts USA; ^3^ Massachusetts General Hospital Biostatistics Boston Massachusetts USA; ^4^ Department of Pathology Massachusetts General Hospital Boston Massachusetts USA; ^5^ Harvard‐MIT Division of Health Sciences and Technology (HST) Boston Massachusetts USA

**Keywords:** B‐mode OCT, endoscopic probe, M‐mode OCT

## Abstract

**Background/Objectives:**

Optical coherence tomography (OCT) uses low coherence interferometry to obtain depth‐resolved tissue reflectivity profiles (M‐mode) and transverse beam scanning to create images of two‐dimensional tissue morphology (B‐mode). Endoscopic OCT imaging probes typically employ proximal or distal mechanical beam scanning mechanisms that increase cost, complexity, and size. Here, we demonstrate in the gastrointestinal (GI) tracts of unsedated human patients, that a passive, single‐fiber probe can be used to guide device placement, conduct device−tissue physical contact sensing, and obtain two‐dimensional OCT images via M‐to‐B‐mode conversion.

**Materials and Methods:**

We designed and developed ultrasmall, manually scannable, side‐ and forward‐viewing single fiber‐optic probes that can capture M‐mode OCT data. Side‐viewing M‐mode OCT probes were incorporated into brush biopsy devices designed to harvest the microbiome and forward‐viewing M‐mode OCT probes were integrated into devices that measure intestinal potential difference (IPD). The M‐mode OCT probe‐coupled devices were utilized in the GI tract in six unsedated patients *in vivo*. M‐mode data were converted into B‐mode images using an M‐to‐B‐mode conversion algorithm. The effectiveness of physical contact sensing by the M‐mode OCT probes was assessed by comparing the variances of the IPD values when the probe was in physical contact with the tissue versus when it was not. The capacity of forward‐ and side‐viewing M‐mode OCT probes to produce high‐quality B‐mode images was compared by computing the percentages of the M‐to‐B‐mode images that showed close contact between the probe and the luminal surface. Passively scanned M‐to‐B‐mode images were qualitatively compared to B‐mode images obtained by mechanical scanning OCT tethered capsule endomicroscopy (TCE) imaging devices.

**Results:**

The incorporation of M‐mode OCT probes in these nonendoscopic GI devices safely and effectively enabled M‐mode OCT imaging, facilitating real‐time device placement guidance and contact sensing in vivo. Results showed that M‐mode OCT contact sensing improved the variance of IPD measurements threefold and side‐viewing probes increased M‐to‐B‐mode image visibility by 10%. Images of the esophagus, stomach, and duodenum generated by the passively scanned probes and M‐to‐B‐mode conversion were qualitatively superior to B‐mode images obtained by mechanically scanning OCT TCE devices.

**Conclusion:**

These results show that passive, single optical fiber OCT probes can be effectively utilized for nonendoscopic device placement guidance, device contact sensing, and two‐dimensional morphologic imaging in the human GI tract in vivo. Due to their small size, lower cost, and reduced complexity, these M‐mode OCT probes may provide an easier avenue for the incorporation of OCT functionality into endoscopic/nonendoscopic devices.

## INTRODUCTION

Optical coherence tomography (OCT) is an optical imaging technique that provides high‐resolution cross‐sectional images of tissue.^1^, ^2^ Endoscopic OCT imaging has been applied in human luminal organs such as the gastrointestinal (GI) tract, airway, cardiovascular system, and auditory canal.^3^, ^4^, ^5^, ^6^, ^7^, ^8^, ^9^ Many luminal endoscopic OCT probes reflect light to the side; mechanical circumferential beam scanning is used to reconstruct a cross‐sectional image. Helical scanning is performed by translating the probe along the organ's axis while the beam rotates, creating three‐dimensional images of luminal organs.^10^ Circumferential beam scanning is typically accomplished using either a distal actuator (e.g., micromotor) that circumferentially scans a reflector that redirects the optical beam,^11^ or a proximal rotary junction that spins the entire OCT probe via a torque coil or driveshaft.^12^ For both cases, the rotating element is enclosed within a transparent sheath to protect the probe and facilitate constant velocity rotation.

Linear beam scanning for OCT imaging in luminal organs has been achieved by axial translation of an OCT probe by a linear actuator placed outside the body mechanically coupled to a translatable wound stainless‐steel coil.^13^, ^14^ Miniaturized electromechanical and pneumatic units can also be placed at the distal end of OCT probes to achieve linear lateral scanning as first reported by Feldchetein et al. and Sergeev et al.^15^, ^16^, ^17^

An alternative approach for endoscopic beam scanning induces mechanical motion of the tip of an optical fiber. One example is scanning fiber endoscopy which deflects an optical fiber's tip using an integrated piezo‐tube scanner within the probe.^18^, ^19^, ^20^, ^21^ In another fiber deflection scheme, an electromagnetic coil is placed at the distal end of the probe to actuate and scan the fiber and thus the optical beam by changing the magnetic field.^22^

Incorporating distal actuators or torque coils within the endoscopic OCT device increases the size of the probe (approximately > 1 mm for distal motors,^23^ >300 µm for torque coils^24^), which may be undesirable for certain applications where size affects safety or maneuverability. Mechanical scanning also increases complexity, potentially making these probes more difficult to manufacture. The need to incorporate rotary junctions, drive cables, and/or micromotors in mechanical scanning probes or systems increases cost. These drawbacks may hinder the incorporation of OCT functionality into certain endoscopic devices that would otherwise benefit from the additional guidance and complementary tissue morphological information that OCT affords.

A nonscanning form of OCT known as M‐mode OCT offers an avenue for achieving OCT imaging without the need for scanning actuators or torque coils. Shin et al.^25^ demonstrated an M‐mode OCT probe for guiding a needle for conducting surgery on the eye. M‐mode probes can also be manually scanned over a tissue surface to create a two‐dimensional image. Ahmad et al., Liu et al., Wang et al., and Marques et al. proposed and refined an algorithm that compares adjacent A‐lines of an image acquired by manually scanned probes. This algorithm retained multiple dissimilar M‐mode OCT A‐lines, determined by thresholding cross‐correlation maxima, to compile B‐scans.^26^, ^27^, ^28^, ^29^ Lee et al.^30^ developed a nonscanning, ultra‐thin OCT probe (OD: 160 µm), used the algorithm proposed by Ahmad et al.,^26^ and captured B‐mode images of a rabbit's trachea and lung ex vivo. To our knowledge, such M‐to‐B‐mode conversion has not yet been reported in human internal luminal organs *in vivo*.

In this paper, we demonstrate side‐ and forward‐viewing, miniature, passively scanned M‐mode OCT probe configurations and demonstrate their utility for guiding GI tract nonendoscopic device placement, contact sensing, and two‐dimensional imaging in living human patients.

## MATERIALS AND METHODS

### M‐mode OCT probe configurations

Passively scanned M‐mode OCT probes were incorporated in intestinal potential difference (IPD)^31^ and brush sampling devices that do not intrinsically employ image guidance. These probe‐coupled devices were introduced into the small intestine via a custom‐fabricated, guide tube termed a transnasal introduction tube (TNIT).^32^ The TNIT is comprised of polyurethane material and has a length of 1.2 m, an outer diameter (OD) of 2.3 mm, and an inner diameter (ID) of 1.6 mm, similar in size and composition to feeding tubes such as nasogastric or nasojejunal tubes.^32^ The introduction process involved inserting the TNIT through the nose of an unsedated subject, deploying the TNIT into the small intestine, and then threading the IPD and brush devices through the TNIT's lumen until their active ends protruded beyond the TNIT, at which point IPD measurements or brush samplings were taken.

**Figure 1:**
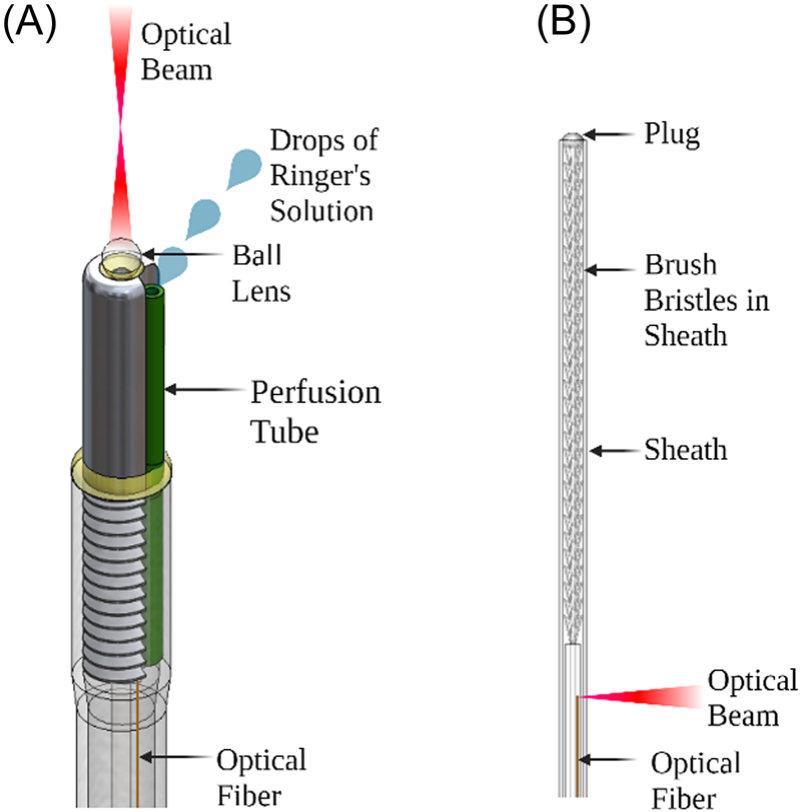
(A) M‐mode OCT probe embedded in an intestinal potential difference (IPD) device. The forward‐viewing probe contains a single‐mode fiber (250 µm diameter) terminated by a distal ball lens. (B) Side‐viewing M‐mode OCT probe, comprising only an angle‐polished fiber (80 mm diameter), attached to an endoscopic sampling brush device. OCT, optical coherence tomography.

The IPD device, with an outer diameter (OD) of 1.2 mm and a length of 1.2 m (Figure 1A),^31^ measures transepithelial voltage as a proxy of small intestinal permeability.^33^ Since the IPD probe was introduced via the TNIT into the small intestine without the aid of a forward‐viewing white light endoscope and the probe requires mucosal contact to obtain accurate measurements, it was critical that the probe contained a contact‐sensing mechanism. The passive M‐mode OCT probe embedded in the IPD device shown in Figure 1A addressed this need. The integrated, forward‐viewing M‐mode OCT probe guided and focused light onto the mucosa and collected light scattered back from the tissue, sending it to an OCT system placed outside the body.

The second implementation was a single‐mode fiber attached to a brush sampling device. The fiber was polished at its distal end to a 45°angle such that the angle of incidence of the beam at the polished surface was greater than the critical angle, reflecting light in a direction that was perpendicular to the fiber's axis (Figure 1B).^34^ The brush was introduced into the GI tract via the TNIT device in the same manner as the IPD device. The addition of the M‐mode OCT probe to the brush helped to discern when it was out of the TNIT and in contact with the mucosa. Once brush placement was confirmed with the aid of M‐mode OCT imaging, it was deployed, and brushing was initiated to collect biomass (cells, mucus, and microbiota). After sampling, the brush was retracted into its sheath to avoid contamination when retracted back through the TNIT.

### OCT imaging system and A‐line rate

**Figure 2:**
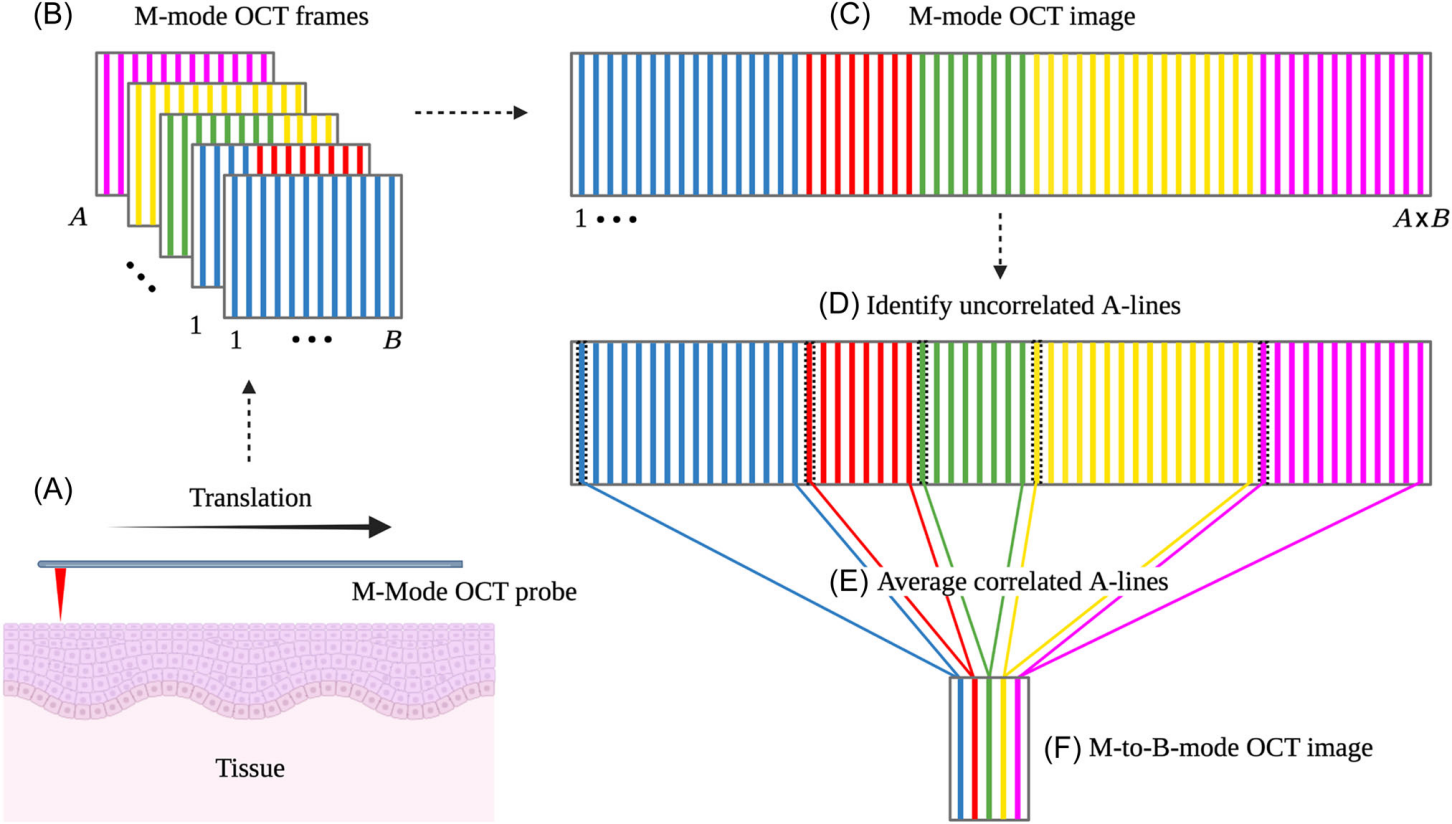
Schematic for acquiring M‐mode OCT frames and converting them to an M‐to‐B‐mode OCT image. (A) M‐mode OCT frames were grabbed as the M‐mode OCT probe was translated over the tissue. The M‐mode OCT frames (B) were concatenated to create one M‐mode OCT image (C). (D) Sequential uncorrelated A‐lines were identified in the M‐mode OCT image. (E) Correlated A‐lines between successive uncorrelated A‐lines were averaged to create one unique A‐line. (F) These averaged A‐lines were placed side‐by‐side to create an M‐to‐B‐mode image. OCT, Optical coherence tomography. Figure created with BioRender. com

The OCT system comprised an Axsun OCT engine (Excelitas Technologies), containing a 1310 ± 75nm swept source (SS) laser, a dual balanced photodetection unit, a field‐programmable gate array (FPGA) module, and a Mach–Zehnder interferometer for k‐clock generation.^35^ The system's A‐line rate was 100 kHz (i.e., 100,000 A‐lines per second). M‐mode frames (Figure 2B, 2560 A‐lines) were transferred across an ethernet bus and stored in real time in a compressed format in a portable compact OCT imaging system.^35^ After the procedure, M‐mode frames were transferred to a desktop computer where they were converted to M‐to‐B‐mode OCT images offline.

### IPD system

The IPD system consisted of an isolation headstage (ISO‐Z, CWe Inc.), a bioamplifier (BMA‐200, CWe Inc.), analog‐to‐digital (A/D) converter (Power Lab 4/26; AD Instruments Inc.), and a computer.^32^ The IPD data signal was detected via an isolation headstage, amplified (10×), digitized using an A/D converter, and recorded using LabChart software (ADInstruments Inc.) at 1000 samples/second.

### OCT tethered capsule endomicroscope (TCE)

The OCT TCE device was comprised of a flexible 1‐mm‐diameter tether, containing a single‐mode optical fiber and electrical wires, connected to an 8−11 mm × 25 mm transparent capsule.^35^ In the capsule, the optical fiber was terminated by a ball lens; converging light from the ball lens was reflected off a micromotor‐driven prism, coming to a 35‐µm‐diameter (FWHM) focus approximately 1 mm from the capsule's outer surface.^35^ The motor in the TCE device rotated at 40 Hz. The TCE device was connected to the same OCT system as that utilized for M‐mode OCT imaging here. After the TCE device was swallowed, it obtained cross‐sectional and three‐dimensional OCT B‐mode images from the upper GI tract in unsedated patients.^36^

### M‐mode to B‐mode conversion algorithm

A schematic of the M‐mode OCT frame acquisition and M‐to‐B‐mode OCT image conversion process is shown in Figure 2. Multiple M‐mode frames were recorded as the M‐mode OCT probe scanned across the tissue (Figure 2A,B). Once the M‐mode OCT frames were collected, *A* M‐mode frames were combined into a single M‐mode OCT image consisting of *A* × *B* A‐lines (Figure 2C), where *B* was the number of A‐lines in an M‐mode frame (2560 A‐lines). Then, a normalized cross‐correlation between zero‐meaned adjacent A‐lines was performed in the Fourier domain, returning a cross‐correlation coefficient *ρ*. *ρ* was subsequently compared to an empirical threshold *ρ*_th_. If *ρ* > *ρ*_th_, the A‐lines were considered correlated. If *ρ* ≤ *ρ*_th_, the two A‐lines were considered uncorrelated (Figure 2D). If the A‐lines were considered correlated, the next A‐line was used for cross‐correlation until an uncorrelated value for *ρ* (*ρ* ≤ *ρ*_th_) was returned. This process was repeated for all uncorrelated A‐lines in the M‐mode OCT image. M‐mode OCT image column indices for consecutive uncorrelated A‐lines were stored in an array. Using the column index array, a new image (M‐to‐B‐mode OCT image) was created such that each consecutive M‐to‐B‐mode image A‐line corresponded to the average of all correlated M‐mode OCT image A‐lines (Figure 2E,F).

### Clinical studies

**Figure 3:**
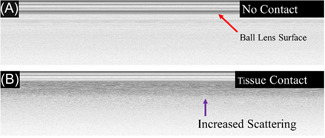
(A) M‐mode OCT image using an IPD probe that was not in contact with the mucosa. (B) M‐mode OCT image obtained with the IPD probe in contact with mucosa as evidenced by the increased tissue light scattering adjacent to the ball lens’ surface. IPD, intestinal potential difference; OCT, optical coherence tomography.

**Figure 4:**
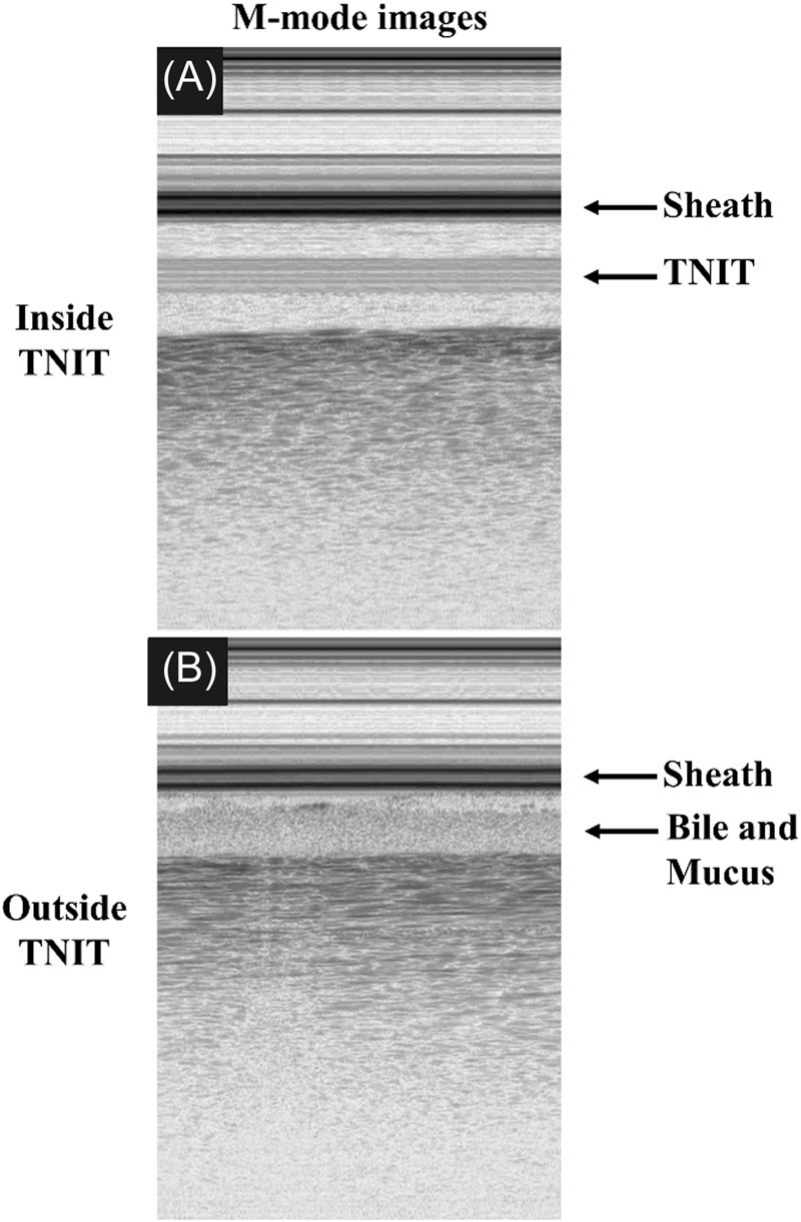
(A) M‐mode OCT images obtained from a patient's duodenum in vivo. When the brush was inside the TNIT, the image showed reflectance coming from the TNIT's wall. (B) When the brush was outside the TNIT, bile/mucus and tissue were visualized, but no such reflectance from the TNIT was seen. OCT, optical coherence tomography; TNIT, transnasal introduction tube.

A total of six patients were enrolled, three (one female, two males) for IPD (IRB no: MGH‐2020P000158) and three (all female) for brush (IRB no: MGH‐2020P000165) at the Massachusetts General Hospital (MGH). After nonendoscopic introduction of the TNIT into the duodenum of unsedated study subjects, the IPD probe and brush devices were manually inserted through the lumen of the TNIT at a speed of ~5 mm/second until the distal end of the devices were outside the TNIT (Figure 3B). Once contact between the device and the tissue was confirmed using real‐time M‐mode OCT imaging (e.g., Figure 4B), brushing or IPD were performed. After brushing or taking IPD measurements, the devices were pulled back through the GI tract at a rate of approximately 5 mm/second until the distal end of the device and the TNIT were at the proximal esophagus. M‐mode OCT frames were recorded during the probe's insertion, brush sampling/IPD measurement, and during the controlled pullback. The frame numbers corresponding to these different phases of the procedure were recorded and used to demarcate regions of interest for subsequent M‐ to B‐mode conversion.

### Data analysis

The capacity of the M‐mode OCT probes to sense physical contact was determined by evaluating the variances of IPD values when M‐mode OCT showed that the IPD was in direct contact with the tissue versus when it was not. The variances of the IPD signal with the probe in direct contact and not in direct contact with the tissue were compared across all the subjects for a duration of 15 minutes using Levene's test. We also compared the capability of side‐viewing (brush) and forward‐viewing (IPD) M‐mode OCT probes to provide high‐quality M‐to‐B‐mode imaging. Since good quality M‐to‐B‐mode imaging requires that the M‐mode OCT probe be close to the luminal surface, the metric of comparison was the percentage of the M‐to‐B‐mode images where the luminal surface was within a fixed distance (100 µm) from the reflection from the M‐mode OCT probe's last optical interface. This “proximity” metric was obtained by calculating the fraction of A‐lines whose OCT signal value at 100 μm from the probe surface was above a fixed threshold (1 SD below mean) in the M‐to‐B‐mode images obtained by the side‐ and forward‐viewing probes. The proximity metric was determined from M‐to‐B‐mode images of the entire esophagus for the two probe configurations in all three patients. Side‐ and forward‐viewing M‐mode OCT probe proximity metrics were compared using a one‐sided Student's *t* test. For all analyses, a *p* value of <0.05 was considered statistically significant.

OCT‐TCE images of the esophagus, stomach, and duodenum were obtained and qualitatively compared with M‐to‐B‐mode images from the same organs, assessing known microscopic morphology based on prior criteria validated in previous OCT histopathologic correlative and case studies.^37^, ^38^, ^39^

## RESULTS

IPD (*n* = 3) and brush sampling devices (*n* = 3) possessing passive M‐mode OCT probes were successfully deployed in unsedated subjects via TNIT introduction. The M‐mode OCT imaging procedures took an average of 4.3 ± 0.47 minutes. No complications or adverse events occurred during the procedures.

Once the IPD or brush probe was outside the TNIT, M‐mode OCT imaging was able to determine when the device was in contact with the mucosa, which is critical for the proper operation of these nonendoscopic measurement/sampling tools. Figure 3A shows an M‐mode OCT image of the IPD probe in the TNIT. The straight dark line represents the M‐mode OCT probe's surface. Figure 3B shows an M‐mode OCT image of the IPD's M‐mode OCT probe in contact with the duodenal mucosa as indicated by the ~1‐mm‐thick scattering layer immediately adjacent to the reflectance from the ball lens’ surface. The clear difference between contact and noncontact images made it easy to visibly confirm tissue contact before initiating IPD measurements. Voltage measurements taken with the nonendoscopic IPD probe in direct contact with the duodenum (mean −11.45± 4.45 mV) were consistent with those obtained endoscopically in prior IPD studies.^40^

M‐mode OCT imaging was helpful in determining when to stop advancing the IPD and brush sampling probes within the TNIT guide. As can be seen in Figure 3, reflectance from the TNIT can be clearly visualized in the M‐mode OCT images when the device was within this introduction tube (Figure 4A). As the device was pushed out of the end of the TNIT, this reflectance disappeared (Figure 4B), notifying the operator to no longer advance the device and initiate taking IPD measurements or brush samples.

**Figure 5:**
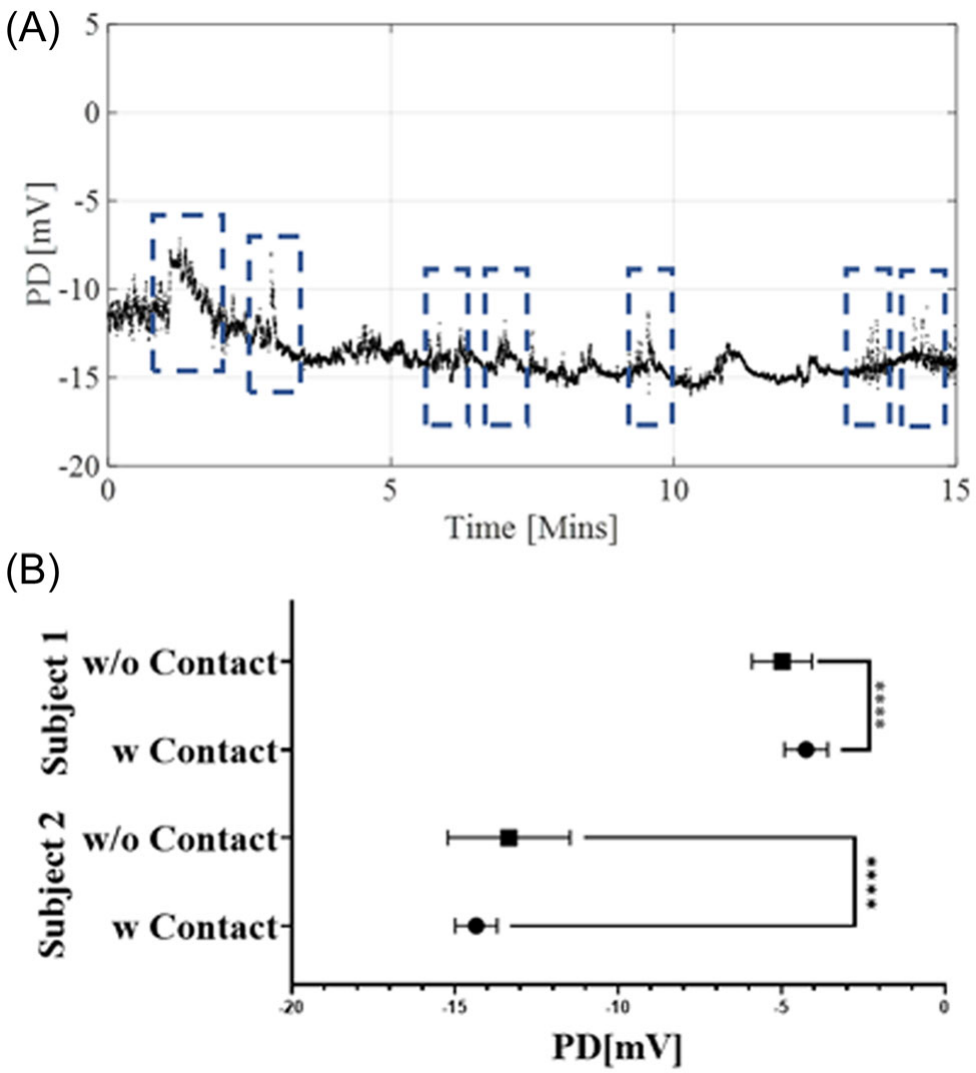
(A) IPD values measured using a forward‐viewing IPD probe in a patient's small intestine with regions of no contact, determined by M‐mode OCT imaging, indicated by the dashed rectangles. (B) Graph showing that the variance in IPD values was greater when the IPD probe was not in contact with the intestinal mucosal surface, as determined by M‐mode OCT imaging. Error bars denote standard deviation. IPD, intestinal potential difference; OCT, optical coherence tomography.

The importance of M‐mode OCT image determination of device‐tissue coupling can be clearly shown by studying IPD measurement variance with and without tissue contact. IPD values and OCT images from two out of three patients were analyzed; data from one of the patients were excluded due to electrical noise in a faulty reference probe. Figure 5A shows IPD values obtained from a patient's (Subject 2) duodenum. Areas where the probe was not in contact with the intestinal mucosa, determined by M‐mode OCT, are indicated by dashed rectangles. The IPD variance when the probe was in direct contact with the mucosa was 0.85 mV versus 0.44 mV when not in direct contact for Subject 1 (*p* < 0.0001). For Subject 2, the IPD variance was 3.50 mV when the probe was not in physical contact with the mucosal surface as opposed to 0.42 mV when it was in direct contact (*p* < 0.0001). These results showed a significantly lower signal variation and thus a more reliable IPD measurement when the device was in physical contact with the mucosal surface, as determined by M‐mode OCT imaging.

**Figure 6:**
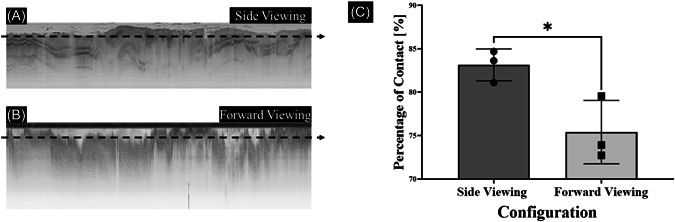
Representative M‐to‐B‐mode images of the esophagus obtained in vivo with the side‐viewing M‐mode imaging brush (A) and the forward‐viewing M‐mode imaging IPD probe (B). Dotted lines represent the depth locations where OCT signal values were measured to ascertain % contact for good M‐to‐B‐mode imaging for both probe configurations. (C) Plot showing that the side‐viewing brush probe was in contact with the mucosal surface for a greater percentage of M‐to‐B‐mode images (*p < 0.05). Error bars denote standard deviation. IPD, intestinal potential difference; OCT, optical coherence tomography.

The proximity of the probe to the tissue also affects M‐to‐B‐mode imaging quality. The potential of side‐ and forward‐viewing M‐mode OCT probes to acquire good M‐to‐B‐mode OCT images, determined by the percentage of the images where the tissue was within a close distance to the probe, was significantly higher for the side‐viewing (brush) probe (83.14 ± 1.46%), compared to the forward‐viewing (IPD) probe (73.94 ± 1.21%) (*p* = 0.0305) (Figure 6C). Supporting this result, the M‐to‐B‐mode images for the side‐viewing configuration were qualitatively superior to those obtained by forward‐viewing M‐mode OCT probes (Figure 6A,B).

**Figure 7:**
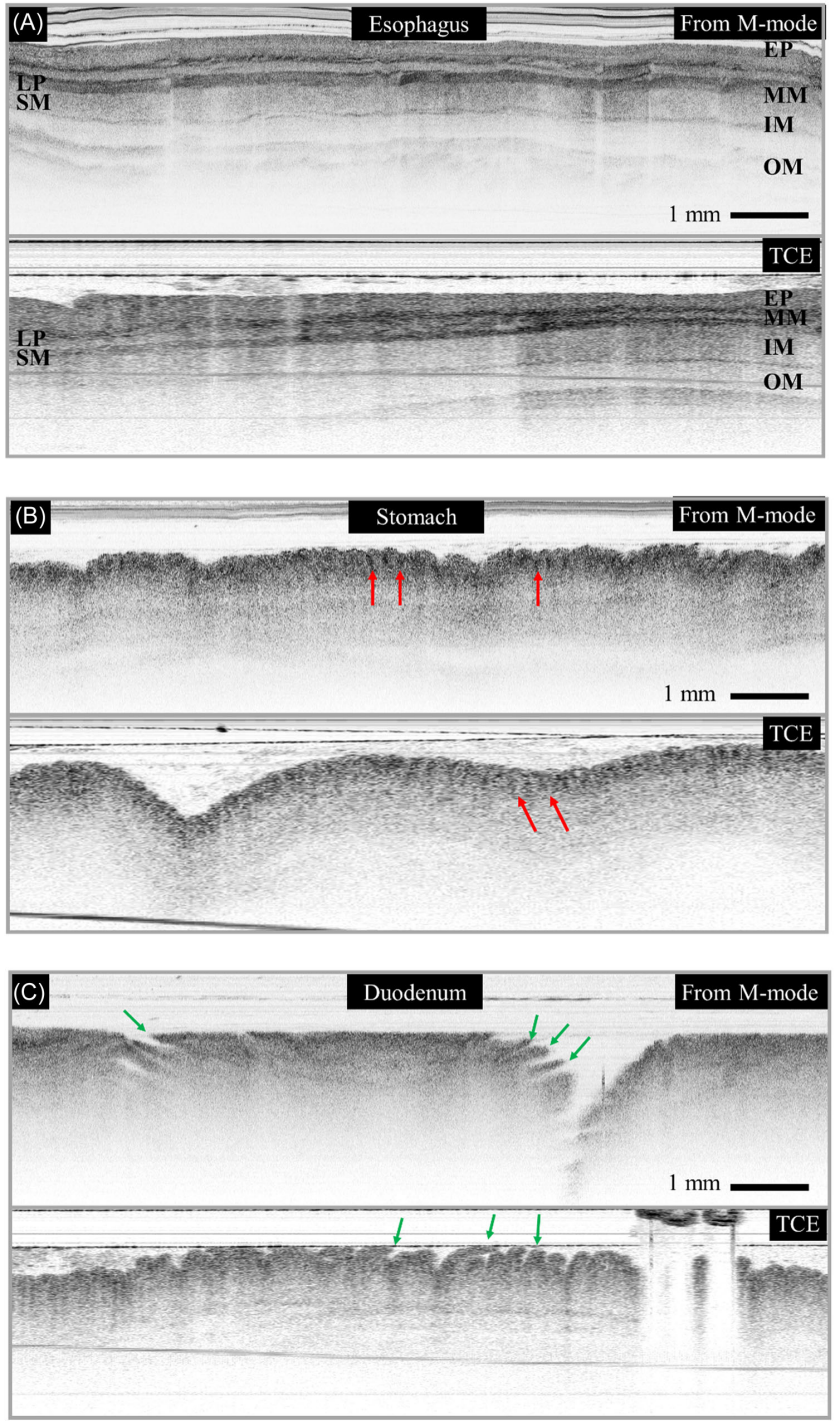
OCT images acquired from the upper gastrointestinal tracts of unsedated subjects, in vivo. (A) An M‐to‐B‐mode images acquired from the esophagus using the side‐viewing M‐mode imaging brush and a B‐mode OCT image of the esophagus acquired using the mechanically scanning tethered capsule endomicroscopy (TCE) device. The epithelium (EP), lamina propria (LP), submucosa (SM), muscularis mucosa (MM), inner muscle (IM), and outer muscular layers can be visualized in the M‐to‐B‐mode OCT image, consistent with the layers observed when using the TCE device. (B) M‐to‐B‐mode OCT image of the stomach obtained with the side‐viewing M‐mode imaging brush and a B‐mode OCT image of the stomach acquired using the mechanically scanning TCE device. Gastric pits (red arrows) were readily visible in the M‐to‐B‐mode frame and in the B‐mode image obtained via the TCE device. (C) M‐to‐B‐mode OCT image of the duodenum captured using the side‐viewing M‐mode imaging brush and the mechanically scanning TCE device. Duodenal villi (green arrows) seen in the M‐to‐B‐mode OCT image were similar to those visualized with the TCE device. OCT, optical coherence tomography.

M‐to‐B‐mode OCT images acquired from patients in vivo using the side‐viewing brush device and B‐mode OCT images obtained in vivo with a mechanically scanning TCE^36^ device are shown in Figure 7. The images acquired with the brush device's M‐mode OCT probe were qualitatively better than those obtained with the mechanically scanning TCE device. Images obtained from the esophagus (Figure 7A) enabled clear visualization of the epithelium, lamina propria, submucosa, muscularis mucosa, inner muscle, and outer muscle layers, equivalent or superior to images of the same acquired with the OCT TCE device (Figure 7A‐TCE). The M‐to‐B‐mode OCT image acquired in the stomach showed gastric pits (Figure 7B), which were similar in appearance but clearer than those seen in images obtained with an OCT TCE device (Figure 7B‐TCE). Both devices enabled clear visualization of duodenal villi (Figure 7C).

## DISCUSSION

In this paper, we have demonstrated the use of passively scanned M‐mode OCT probes for imaging the human GI tract in vivo. We have shown that these probes can be utilized for guiding the placement of nonendoscopic GI devices so that procedures such as IPD measurement and brush sample collection can be optimally conducted. M‐mode OCT proved to be useful for physical contact sensing, as shown by the lower IPD value variances when the probe was determined to be in direct physical contact. We also found that side‐viewing probes were better for M‐to‐B‐mode conversion imaging owing in part to the increased percentage of time that these probes were close to the luminal surface. Finally, our results showed that the M‐mode images obtained with the forward‐ and side‐viewing probes can be converted to B‐mode OCT images that are of equal or superior quality to those obtained with more complex mechanical beam scanning mechanisms.

Physical contact is an essential component required for the adequate function of many GI devices. As demonstrated here, this direct contact improves measurement reliability (e.g., statistically significant lower variance in IPD measurements). It is also likely to be critical for any type of nonendoscopic tissue sampling device (e.g., brush or forceps biopsy) where the biopsy tool needs to be in direct contact with the mucosa for adequate samples to be acquired. Such physical sensing for nonendoscopic devices is now possible by simply adding a cleaved fiber to the device and performing real‐time M‐mode OCT imaging.

Another benefit of M‐mode sensing is apparent when using a guide tube (e.g., TNIT) to deliver a device to the GI tract blindly, without endoscopic guidance. To ensure the device is located on the mucosa, it is critical to know when the device has exited the guide tube and is ready to take the measurement/sample. The M‐mode OCT probes described here can be used to make this assessment in real time (Figure 4), allowing the operator to know when to stop advancing the device in the guide tube, when the device's active region is on the targeted tissue, and thus when the measurement can be taken, or the intervention performed.

B‐mode conversion of M‐mode images acquired by these probes also potentially has a significant impact. Previously, when a measurement or tissue sample was taken from the GI tract, there was no record of what this tissue looked like in the living patient before the measurement or perturbation. With the addition of the M‐mode OCT probes to devices, the M‐to‐B‐mode images provide such a record, enabling new relationships to be investigated. For example, since this technology enables M‐to‐B‐mode images and IPD to be acquired from the same location, we can now study the relationships between tissue morphology and intestinal permeability in vivo.

M‐to‐B‐mode images were generally qualitatively superior to images acquired using a mechanically scanning TCE device (Figure 7). One reason for this difference in image quality may be due to a higher lateral sampling density, as the M‐mode OCT probes typically scan over the tissue at a much slower rate than mechanically scanning TCE devices. Side‐viewing images were qualitatively clearer and showed more detail compared to images obtained with the forward‐viewing probes (Figure 6A,B). This difference may be due to decreased motion artifact, as side‐viewing M‐mode OCT probes lie on the luminal surface and are thus well coupled to the tissue that they are imaging, whereas forward‐viewing M‐mode OCT probes are likely less stable when imaging.

While there are many advantages to M‐mode OCT imaging, these probes cannot be used for helical scanning, limiting their utility for volumetric OCT imaging of large portions of luminal organs.^36^, ^41^ It also may be difficult to discern which path (straight line or zigzag line) the probe takes, as the relationship between the proximal and distal ends of a 1.2‐m‐long flexible probe within the luminal organ is poorly defined in vivo.

In summary, we have shown that passive, single‐fiber, M‐mode OCT probes can be utilized for physical contact sensing and two‐dimensional, depth‐resolved microscopic imaging in the upper GI tract of living human patients. Results demonstrated that this technology improves measurement reliability, provides essential information on the position of devices within guide tubes, and generates B‐mode OCT images, enabling tissue morphology in the esophagus, stomach, and duodenum to be visualized in vivo. The small size and simplicity of these passive, single‐fiber probes and the important and in some cases essential functionality that they provide make them attractive as an adjunct to a variety of different GI tract measurement and sampling devices that do not rely on endoscopy for guidance.

## CONFLICT OF INTEREST

Dr. Tearney has financial/fiduciary interest in SpectraWave, a company developing an OCT‐NIRS intracoranary imaging system and catheter. His financial/fiduciary interest was reviewed and is managed by the Massachusetts General Hospital and Mass General Brigham in accordance with their conflict of interest policies.
